# Supplementary material for: Trends in pediatric cochlear implants: The dual impact of COVID-19 and Lebanon’s crisis
Source: PLoS One. 2025 Sep 25;20(9):e0331234. doi: 10.1371/journal.pone.0331234 (PMC12463290; doi:10.1371/journal.pone.0331234)
Supplement: Attachment — Submitted filename: PONE Recommendations.docx [file pone.0331234.s002.docx]

PONE

76 typically ranges from $15,000 to $40,000 [10]. This Fig does not account for ongoing expenses

88 procedures, were deprioritized due to strain on healthcare systems. As sources were redirected to

89 urgent care, many CI were postponed, affecting patients who rely on these procedures for hearing

92 Therefore, a methodological evaluation can be used to raise awareness and to benefit from 93 governments support.

226 and Latin America have performed CI, they have been faced with numerous challenges [17], [18],
